# Gene-Environment Interaction in a Conditional NMDAR-Knockout Model of Schizophrenia

**DOI:** 10.3389/fnbeh.2018.00332

**Published:** 2019-01-10

**Authors:** Alexei M. Bygrave, Simonas Masiulis, Dimitri M. Kullmann, David M. Bannerman, Dennis Kätzel

**Affiliations:** ^1^Department of Experimental Psychology, Medical Sciences Division, University of Oxford, Oxford, United Kingdom; ^2^Institute of Neurology, University College London, London, United Kingdom; ^3^Institute of Applied Physiology, University of Ulm, Ulm, Germany

**Keywords:** schizophrenia, gene-environment interaction, NMDAR-receptor hypofunction, interneurons, risk factors

## Abstract

Interactions between genetic and environmental risk factors take center stage in the pathology of schizophrenia. We assessed if the stressor of reduced environmental enrichment applied in adulthood provokes deficits in the positive, negative or cognitive symptom domains of schizophrenia in a mouse line modeling NMDA-receptor (NMDAR) hypofunction in forebrain inhibitory interneurons (*Grin1*^Δ*Ppp1r2*^). We find that *Grin1*^Δ*Ppp1r2*^ mice, when group-housed in highly enriched cages, appear largely normal across a wide range of schizophrenia-related behavioral tests. However, they display various short-term memory deficits when exposed to minimal enrichment. This demonstrates that the interaction between risk genes causing NMDA-receptor hypofunction and environmental risk factors may negatively impact cognition later in life.

## Introduction

In addition to prenatal influences (van Os et al., 2010), environmental factors like drug abuse, social stressors or urbanicity can determine severity of symptoms and treatment outcome in patients with *established* schizophrenia (Parker and Hadzi-Pavlovic, 1990; Linszen et al., 1997; Corcoran et al., 2002; Peterson and Docherty, 2004; van Os et al., 2010; Réthelyi et al., 2013). Interestingly, improvement in the quality of life by enhancing leisure activity or physical exercise (Gorczynski and Faulkner, 2010; Carta et al., 2014; Rosenbaum et al., 2014; Dauwan et al., 2016; Firth et al., 2017), but also psychosocial treatment (Penn and Mueser, 1996) may improve symptoms and relapse probabilities. Rodent models of schizophrenia have mostly replicated the combination of genetic aberrations and prenatal insults (Nagai et al., 2011; Lipina et al., 2013), but the influence of environmental stressors later in life in animals carrying genetic risk factors has hardly been explored.

Therefore, we investigated gene-environment interactions in a mouse model with early-onset conditional ablation of NMDA-receptors (NMDAR) in forebrain inhibitory interneurons of which over 70% were reported to be parvalbumin-positive, while the remainder express mostly reelin or neuropeptide-Y (Belforte et al., 2010). Previous studies have demonstrated an exacerbation of correlates of positive, negative and cognitive symptoms of schizophrenia by social-isolation stress in these mice (Belforte et al., 2010; Jiang et al., 2013a,b). However, long-term social-isolation starting during development or in adulthood is quite a severe stressor in social animals producing multiple changes in psychiatrically relevant behaviors, genes and neuronal structure on its own (Kercmar et al., 2011; Ieraci et al., 2016; Castillo-Gómez et al., 2017)—thus potentially not quite modeling changes in quality of life in adult humans. Therefore, we asked, whether a milder stressor, namely reduced environmental enrichment may interact with the genetic predisposition to establish symptoms of schizophrenia in this model of NMDAR-hypofunction.

## Methods

### Subjects and Cage Enrichment

To replicate a mouse line with conditional ablation of NMDARs in forebrain interneurons (Belforte et al., 2010), we crossed a BAC-transgenic line in which *Cre*-recombinase is driven by the promoter of the *Ppp1r2* gene (Belforte et al., 2010), to a line where the constitutive NMDA receptor subunit 1 (*Grin1*) contains two lox-sites at a short distance from each other to facilitate *Cre*-dependent ablation (termed *Grin1*^Δ*Ppp1r2*^ knockouts or KO below; Korotkova et al., 2010; Bygrave et al., 2016). *Cre*-negative floxed-*Grin1* littermates served as controls (Ctrl; see Supplementary Methods for all methodical details on the animals and behavioral assessment described below).

Male mice were bred and raised in individually ventilated cages (IVCs) with high levels of enrichment using sizzle nest, a card board house and a cardboard tube (Datesand, Manchester, UK). At 4–6 weeks of age one group of mice (*n*: 7 KO, 6 Ctrl) was transferred to IVC-cages with minimal enrichment (reduced environmental enrichment group, RE) featuring only one Nestlet™ cotton pad (Datesand, Manchester, UK), while the other group (*n*: 9 KO, 11 Ctrl) was kept in an highly enriched (HE) environment, consisting of open-top cages filled like the breeding cages (sizzle nest, tube, house) to provide rich tactile, auditory and olfactory stimulation (Supplementary Figure S1). Animals were housed in mixed-genotype groups of 2–5 animals per cage. Six weeks later a test battery assessing mouse correlates of positive, negative and cognitive symptoms of schizophrenia commenced. The average age at testing is mentioned in Supplementary Methods and Supplementary Table S1.

### Behavior

**Figure 1:**
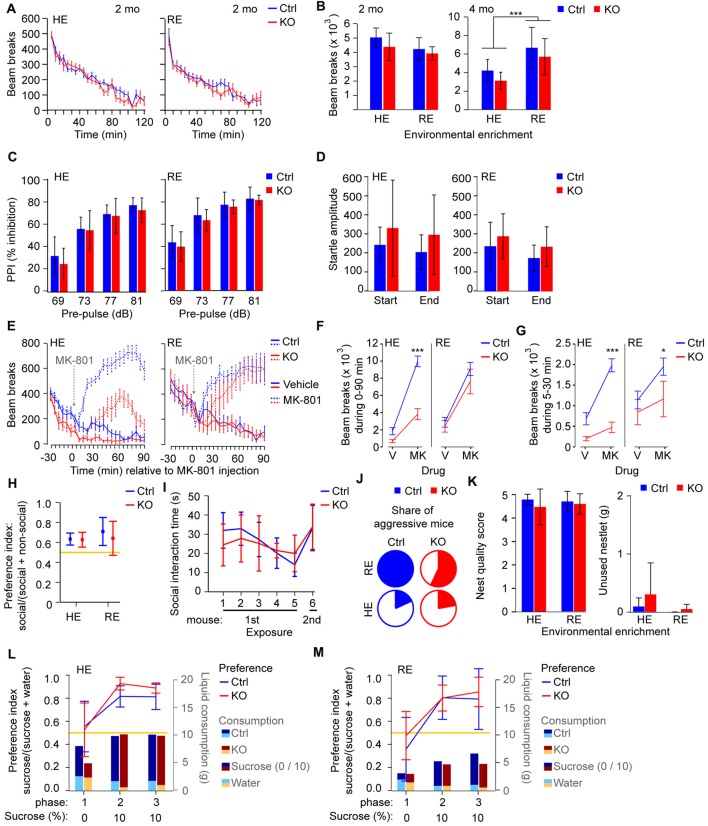
Behavioral correlates of positive and negative symptoms are largely normal in *Grin1*^Δ*Ppp1r2*^ animals irrespective of environmental enrichment. **(A,B)** Novelty-induced hyperlocomotion. **(A)** Average beam-break counts over 120 min displayed in 5 min bins for control (blue) and *Grin1*^Δ*Ppp1r2*^ (red) mice aged 2 months from high (HE) or reduced (RE) environmental enrichment conditions. **(B)** Summed beam breaks during the 120 min session for animals of 2 (left) and 4 months of age (right). **(C)** Average pre-pulse inhibition (PPI) expressed as % of the startle at individual dB-levels of the pre-pulse. **(D)** Average absolute response to the startle-pulse (120 dB) at the start and end of the test sequence, showing mild habituation to the startle-pulse over time. **(E)** Average number of beam breaks over 30 min before and 90 min after injection of vehicle (solid lines) or 0.2 mg/kg MK-801 (at time 0, dashed lines) in 5 min intervals under RE (left) or HE (right) conditions. **(F,G)** Summed beam breaks during the 0–90 min **(F)** and 5–30 min **(G)** period after vehicle/MK-801 injection. **(H)** Non-reciprocal social interaction (3-chamber test) in HE and RE groups with average sociability displayed as a ratio (time in social interaction zone/time in social and non-social interaction zones combined). **(I)** Reciprocal social interaction protocol in the HE cohort with five consecutive exposures to the same stimulus mouse followed by one exposure to a novel mouse. Sociability displayed as the average social interaction time. Data for reciprocal social interaction is only shown for the HE due to aggression in the RE cohort. **(J)** Share of aggressive mice in the HE and RE cohorts shown in color. **(K)** Assessment of nest building with average nest quality score (left) and average unused bedding material (right) quantified. **(L,M)** Assessment of sucrose preference in HE **(L)** and RE **(M)** groups. Average preference for 10% sucrose displayed as a ratio (10% sucrose consumed/total liquid consumed) by the line graph (left axis). Average consumption of 10% sucrose (dark color) and water (bright color) for each group displayed by bar graphs (weight of liquid in grams, g; right axis). In all cases error bars display 95% confidence intervals except in **(A,E–G)** where the SEM is shown for clarity. Data from control mice (Ctrl) are displayed in blue, data from knockouts (KOs) in red. *p < 0.05; ***p < 0.001; simple main effects if shown within HE/RE group, analysis of variance (ANOVA) if shown between HE/RE groups. Yellow lines indicate chance level performance, relating to the left axis.

All behavioral protocols are identical to those used previously for the assessment of conditional NMDAR-KO lines (Belforte et al., 2010; Bygrave et al., 2016) and their details can be found in Supplementary Methods. Analysis was conducted with repeated-measures or univariate analysis of variance (ANOVA), as appropriate, to identify effects of genotype, environment and gene-environment interactions, followed by simple main effects *post hoc* tests. Non-parametrically distributed data (from the nest building test) were analyzed with a Mann-Witney-U test and binary counts of aggressive behavior by Fisher’s exact test. Figure plots show means ±95% confidence intervals drawn symmetrically around the mean, except for Figures 1A,E–G, where SEM are used for clarity. All raw data are available from the corresponding authors (DK) at reasonable request.

## Results

### Rodent Correlates of the Positive and Negative Symptom Domain

Related to the positive symptom domain, we assessed spontaneous novelty-induced hyperlocomotion, pre-pulse inhibition (PPI) and habituation of the startle response. In all three domains, *Grin1*^Δ*Ppp1r2*^ animals appeared normal (*p* > 0.05 for effects of genotype and gene*environment interaction; ANOVA; Figures 1A–D; see Supplementary Table S1 for statistical details on this and all subsequent tests). Enriched environment alone, independent of genotype, led to a significantly lower PPI (*p* = 0.033; Figure 1C) and—at older age—reduced locomotion (*p* < 0.0005; ANOVA; Figure 1B).

Mice in which NMDARs are ablated from parvalbumin-interneurons, including the line used here, have previously been shown to display reduced enhancement of locomotion by the NMDAR-blocker MK-801 (Belforte et al., 2010; Carlén et al., 2012; Bygrave et al., 2016). We demonstrated previously that this effect is due to repeated catalepsy occurring exclusively in these mice, but not in controls, at a dose of 0.2 mg/kg MK-801 for example (Bygrave et al., 2016). We repeated the same experiment with the current cohort (Figure 1E; within-subjects design) and analyzed two measures: the total locomotor-activity throughout the 90 min post-injection period (Figure 1F) and the locomotor-activity specifically during 5–30 min, in which the locomotion-reducing effect is strongest in *Grin1*^Δ*Ppp1r2*^ mice (Figure 1G). As expected, *Grin1*^Δ*Ppp1r2*^ mice showed lower locomotion than controls after MK-801, but this effect was much less evident in the RE group compared to the HE group. We obtained significant effects of genotype (*p* ≤ 0.001), environment (*p* < 0.05), drug (*p* ≤ 0.0005) and a drug-genotype interaction (*p* ≤ 0.002) in both measures, and additional genotype-environment (*p* < 0.05) and drug-genotype-environment (*p* ≤ 0.0005) interactions in the first (0–90 min) measure (repeated-measures ANOVA). This indicates that the level of enrichment modulates the NMDAR-hypofunction-dependent responsiveness to global NMDAR blockade. Also, observation of a subset of these mice revealed catalepsy in the KO mice but not in wild-type controls at this dose, consistent with our previous study (Bygrave et al., 2016).

In the negative symptom domain, we assessed social interaction, nest building and sucrose preference. Non-reciprocal social interaction, measured with the 3-chamber test, was not impaired irrespective of genotype and housing condition (Figure 1H). Likewise, using the reciprocal social interaction protocol applied for the original phenotyping of this line (Belforte et al., 2010), we found no significant impairment in mice from HE cages (Figure 1I). Some mice conducted extended aggressive attacks on the stimulus mice. Intriguingly, this was mainly seen in the RE cohort preventing the analysis of social interaction behavior in this group during the reciprocal test (Figure 1J). However, there were no significant differences in the percentage of mice displaying aggressive behavior across the four subgroups (*p* > 0.1, Fisher’s exact test). Furthermore, we did not observe anhedonia (decreased preference for sucrose) or deficits in nest building in *Grin1*^Δ*Ppp1r2*^ mice in either housing condition (Figures 1K–M).

### Reduced Environmental Enrichment Evokes Cognitive Deficits in *Grin1*^Δ*Ppp1r2*^ Mice

**Figure 2:**
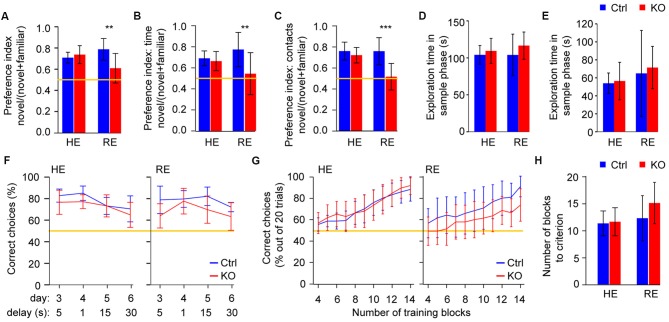
Reduced environmental enrichment induces short-term memory deficits in *Grin1*^Δ*Ppp1r2*^ animals. **(A)** Spatial novelty-preference (SNP) Y-maze test with average novelty preference displayed as a ratio (time in novel arm/time in both choice arms). **(B,C)** Novel object recognition (NOR) with preferences for novel objects displayed as ratios (interaction with novel object/interaction with both objects) calculated using either the total time of interaction **(B)** or the number of contacts **(C)**. **(D,E)** Duration of exploration of the to-be-familiar arm during the sample trial in the SNP Y-maze test **(D)** and of the to-be-familiar object in the sample trial of the NOR test **(E)** during the sample phase of each task. **(F)** Rewarded alternation test of spatial working memory in HE (left) and RE (right) groups. Average performances are displayed as % of correct trials out of 10 trials conducted for each testing condition (delays and trial structure). The data represents averages across the first and the second session (conducted ca. 1 months apart) within each protocol. The first 2 days of initial training in each session are not shown. **(G,H)** Plus-maze assessment of appetitive long-term spatial memory. **(G)** Average % of correct choices made in the last 20 trials (four blocks of five trials each) in HE (left) and RE (right) groups shown across blocks 4–14, and **(H)** average number of training blocks required to reach the criterion (performance level of 17/20, i.e., 85% correct in four consecutive blocks). In **(A–C)** and** (F,G)** the yellow line indicates chance level performance. In all cases error bars display 95% confidence intervals. **p < 0.01; ***p < 0.001; simple main effects.

Deficits of short-term memory which underpin short-term habituation of attention are central to aberrant cognition and salience in schizophrenia (Barkus et al., 2014). We assessed a spatial and an object-related form of short-term memory, using the Y-maze spatial novelty-preference (SNP) test and novel-object recognition (NOR) respectively. In both tests a significant gene-environment interaction (*p* < 0.05, ANOVA) was revealed whereby *Grin1*^Δ*Ppp1r2*^ mice from cages with RE showed impaired short-term memory (*p* < 0.01, simple main effects), while *Grin1*^Δ*Ppp1r2*^ animals from HE cages performed like HE control mice (*p* > 0.5; Figures 2A–C). These differences were not due to different amounts of time exploring the to-be familiar object or spatial locations, respectively, during the sample phases (Figures 2D,E).

To assess spatial working memory, we performed the rewarded alternation (non-matching to place) test on the T-maze using a 3 day training protocol with a delay (intra-trial interval) of 5 s and subsequent challenges with delays of 1, 15 and 30 s. The sequence was repeated 1 month later and the data were averaged within protocols (i.e., for each delay). Across the four protocols (i.e., delays, days 3–6, see Figure 2F) a significant overall impairment was seen in the KOs (*p* < 0.05, repeated-measures ANOVA), but no gene-environment interaction was apparent. However, with all delay protocols, *Grin1*^Δ*Ppp1r2*^ mice still performed above chance levels (see Figure 2F). This suggests that—as shown in other studies with interneuron-specific NMDAR-KO (Carlén et al., 2012; Bygrave et al., 2016) — this line displays a minor working memory deficit. During the initial three training days—but not in later protocols—enrichment itself resulted in significantly better performance, irrespective of genotype (*p* = 0.013, ANOVA; not shown).

We also assessed spatial long-term memory using an appetitively motivated, associative learning paradigm in the plus-maze, but we found no significant effects of genotype, enrichment or any interaction (*p* > 0.1, for either performance over the first 14 training blocks or the number of blocks needed to reach to criterion; Figures 2G,H). This demonstrates that general spatial processing is intact, and therefore does not confound prior spatial short-term memory readouts in these mice.

## Discussion

We found that *Grin1*^Δ*Ppp1r2*^ mice appear largely normal when maintained in enriched environments but display some cognitive deficits, namely spatial and object-related short-term memory impairments, when housed under reduced environmental enrichment.

Also, enrichment strongly modulated the susceptibility of *Grin1*^Δ*Ppp1r2*^ mice to pharmacologically induced NMDAR-hypofunction. In this case, however, it appeared that RE produced a locomotor phenotype in KOs that was more similar to wildtype behavior (i.e., higher locomotion under MK-801), while *Grin1*^Δ*Ppp1r2*^ mice from enriched cages displayed a more pronounced reduction of MK-801-induced locomotion. However, the levels of ambulatory locomotor activity that we observed in Figure 1E likely reflect an interaction between two potentially distinct effects of MK-801—increasing locomotor drive on the one hand, but inducing catalepsy in KOs, on the other. It is possible that the pattern of results that we see in HE and RE mice reflects an enhanced sensitivity to the locomotor promoting effects of MK-801 in the RE mice. The significantly higher spontaneous locomotion in RE-animals in general across our studies (Figures 1B,E–G) is consistent with this possibility and could offset the catalepsy-inducing effect of MK-801 in *Grin1*^Δ*Ppp1r2*^ mice.

Putting our observations into the context of two previous phenotyping studies in this mouse model, one can assume that most of the schizophrenia-related deficits displayed by this line are dependent on some form of environmental stress during some stage of development. For example, impaired spatial short-term memory tested in the Y-maze can be provoked by reduced environmental enrichment (according to our data) but also by long-term social isolation starting at an early age (right after weaning; Jiang et al., 2013b). Deficits in nest-building and anhedonia could not be induced by RE in adulthood in our hands but have been found after long-term social isolation, which may reflect the higher severity of this stressor and/or its earlier onset (Jiang et al., 2013b). The same is likely to be true for reduced sociability which has only been demonstrated in socially isolated *Grin1*^Δ*Ppp1r2*^ mice so far and is not present in mice from our study, irrespective of enrichment (Belforte et al., 2010).

Our results highlight that even seemingly subtle changes to environmental conditions can determine if impairments are apparent in preclinical models of schizophrenia. They furthermore support our previously suggested model that NMDAR-hypofunction in PV-interneurons may constitute just one out of many *risk factors* for schizophrenia (Bygrave et al., 2016). Only its interaction with other risk factors, such as environmental stress or NMDAR-hypofunction on other neurons in the circuit leads to schizophrenia-related deficits. One mechanism as to how environmental stress may interact with the risk factor of NMDAR-hypofunction in interneurons could involve metabolic stress: it has been well documented that reduced environmental enrichment increases oxidative stress levels in rodents (Cechetti et al., 2012; Muhammad et al., 2017) and that NMDAR-hypofunction in PV-interneurons in the mouse line we used in this study renders those interneurons more vulnerable to oxidative stress (Jiang et al., 2013a,b). Finally, for the clinical realm, our data encourages the establishment of a stimulating and stress-free environment to improve symptoms in schizophrenia (Rogers et al., 2017).

## Ethics Statement

This study was carried out in accordance with the recommendations of the “Animal (Scientific Procedures) Act 1986, UK,” and the “Local Ethical Review Committee at the University of Oxford.”  The protocol was approved by the “Home Office of the United Kingdom.”

## Author Contributions

AMB, DMB and DK designed the experiments, analyzed the data and wrote the manuscript. AMB, SM and DK conducted the experiments. DMB and DMK contributed essential resources, and advised on the experimental design and the manuscript.

## Conflict of Interest Statement

The authors declare that the research was conducted in the absence of any commercial or financial relationships that could be construed as a potential conflict of interest.
