# Supplementary material for: Gene-Environment Interaction in a Conditional NMDAR-Knockout Model of Schizophrenia
Source: Front Behav Neurosci. 2019 Jan 10;12:332. doi: 10.3389/fnbeh.2018.00332 (PMC6338026; doi:10.3389/fnbeh.2018.00332)
Supplement: Supplementary file 1 [file Data_Sheet_1.PDF]

Supplementary Information

Gene-environment interaction in a conditional NMDAR-knockout model of schizophrenia

Running title: Environmental enrichment prevents cognitive deficits

Authors:

Alexei M. Bygrave[1] , D.Phil, Simonas Masiulis, D.Phil[1], Dimitri M. Kullmann, D.Phil[2]*, David M. Bannerman, Ph.D.[1]*, Dennis Kätzel, D.Phil[1,2,3]*

[1] Dept. of Experimental Psychology, University of Oxford, Oxford, UK

[2] Institute of Neurology, University College London, London, United Kingdom

[3] Institute of Applied Physiology, Ulm University, Ulm, Germany

* These authors supervised the project equally.

*** Correspondence:**

Dimitri M. Kullmann, Institute of Neurology, University College London, Queen Square, WC1N 3BG, London, United Kingdom, d.kullmann@ucl.ac.uk

David M. Bannerman, Dept. of Experimental Psychology, University of Oxford, Tinsley Bldg., Mansfield Road, OX1 3SR, Oxford, United Kingdom, david.bannerman@psy.ox.ac.uk

Dennis Kätzel, Institute of Applied Physiology, Ulm University, 89081 Ulm, Germany; dennis.kaetzel@uni-ulm.de

Supplementary Figures

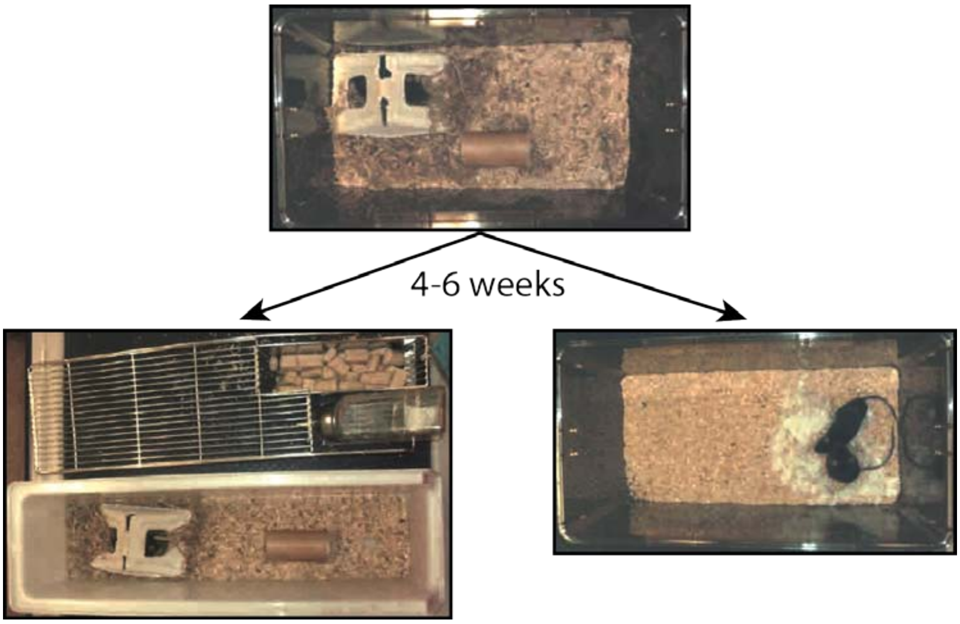

Supplementary Figure 1. Illustration of the enrichment of cages.

Cages in which the mice were bred and raised were individually ventilated and contained saw-dust, sizzle nest, cardboard houses, and a cardboard tube as enrichment (top). Between 4-6 weeks of age mice were either transferred into highly enriched open-top cages (left) containing the same enrichment elements as the breeding cages and allowing richer olfactory and auditory stimulation through the open metal grid (bottom left), or they were maintained in standard (non-enriched) cages which contained only sawdust and one Nestlet cotton pad, which they could rip apart to build a nest (bottom right).

Supplementary tables

Supplementary Table 1

Supplementary Table 1a					ANOVA						S. main effect	
Analysis of behaviour with univariate ANOVA			age		genotype		environment		interaction		genotype	
Behaviour	Tested variable	Fig.	(mo)	df1, df2	F	p	F	p	F	p	RE	HE
Novelty-induced locomotion	Total distance moved	1B	2	1, 29	1.051	0.314	3.353	0.077	0.026	0.874	0.583	0.353
	Total distance moved	1B	4	1, 29	2.599	0.118	16.8	<0.0005	0.008	0.930	0.336	0.186
Pre-pulse inhibition	PPI (69dB)	1C	4	1, 25	0.696	0.412	3.510	0.073	0.026	0.873	0.664	0.447
	PPI (73dB)	1C	4	1, 25	0.539	0.470	3.851	0.061	0.266	0.611	0.422	0.867
	PPI (77 dB)	1C	4	1, 25	0.322	0.576	3.847	0.074	0.043	0.838	0.618	0.782
	PPI (81 dB)	1C	4	1, 25	0.666	0.422	3.978	0.057	0.158	0.695	0.787	0.355
	Habituation	1D	4	1, 25	0.040	0.949	0.553	0.464	0.000	0.992	0.972	0.954
	Startle (Begin)	1D	4	1, 25	0.718	0.405	0.096	0.759	0.253	0.619	0.824	0.304
Non-reciprocal sociability	Preference: interaction time	1I	3	1, 29	0.701	0.409	1.018	0.321	0.493	0.488	0.331	0.915
Sucrose preference	Preference (Day 2)	1M-N	4	1, 29	1.431	0.241	2.339	0.137	1.478	0.234	0.990	0.065
Y-maze SNP	Preference ratio: time	2A	2	1, 29	3.797	0.061	0.273	0.605	7.220	0.012	0.006	0.561
	Exposure time (sample phase)	2D	2	1, 29	1.340	0.257	0.207	0.653	0.221	0.642	0.311	0.580
Novel-object recognition	Preference ratio: time	2B	3	1, 24	8.969	0.006	2.168	0.154	5.776	0.024	0.002	0.629
	Preference ratio: contacts	2C	3	1, 24	10.62	0.003	0.524	0.476	8.442	0.008	0.001	0.773
	Exposure time (sample phase)	2E	3	1, 24	0.009	0.926	3.890	0.061	0.000	0.990	0.96	0.930
T-maze working memory	Score: Training (day 1-3)	2F	2, 3	1, 29	3.990	0.055	6.977	0.013	1.028	0.319	0.532	0.023
	Score: 1s delay (day 4)	2F	2, 3	1, 29	1.995	0.168	0.489	0.490	0.697	0.410	0.713	0.084
	Score: 5s delay (day 3)	2F	2, 3	1, 29	6.015	0.020	3.731	0.063	1.016	0.322	0.034	0.260
	Score: 15s delay (day 5)	2F	2, 3	1, 29	2.084	0.164	0.398	0.533	2.072	0.161	0.076	0.993
	Score: 30s delay (day 6)	2F	2, 3	1, 29	1.872	0.182	0.000	0.994	0.096	0.759	0.290	0.407
Reference memory	Trials to criterion	1H	4	1, 29	1.398	0.247	2.853	0.102	0.907	0.349	0.181	0.856

Supplementary Table 1b				Repeated-measures ANOVA															
Analysis of behaviour with repeated measures ANOVA			age		genotype		environment		interaction		WSF		WSF*env		WSF*geno		WSF*env*geno		
Behaviour	Tested variable (WSF)	Fig.	(mo)	df1, df2	F	p	F	p	F	p	F	p	F	p	F	p	F	p	
PPI	PPI (all 4 dB levels)	1C	4	1, 25	0.773	0.388	5.112	0.033	0.005	0.943	116.5	<0.0005	0.6	0.518	0.179	0.778	0.331	0.662	
	Habituation (Begin vs End)	1D	4	1, 25	1.041	0.317	0.294	0.593	0.265	0.611	8.416	0.008	0.469	0.500	0.062	0.806	0.030	0.864	
Reciprocal sociability	Interaction time (exposures 1-5)	1J	4	1, 14	0.202	0.660	-	-	-	-	4.676	0.008	-	-	1.095	0.368	-	-	
	Interaction time (exposure 1 vs 5)	1J	4	1, 14	0.065	0.803	-	-	-	-	7.050	0.019	-	-	2.557	0.132	-	-	
	Interaction time (exposure 2 vs 5)	1J	4	1, 14	0.015	0.905	-	-	-	-	10.07	0.007	-	-	1.712	0.212	-	-	
Reciprocal social memory	Interaction time (exposure 5 vs 6)	1J	4	1, 14	0.460	0.508	-	-	-	-	21.35	<0.0005	-	-	0.533	0.478	-	-	
Sucrose preference	Preference (all days)	1M-N	4	1, 28	1.131	0.297	1.893	0.180	0.000	0.986	51.17	<0.0005	0.642	0.466	0.054	0.849	1.398	0.250	
	Preference (day 1 vs 2)	1M-N	4	1, 28	0.760	0.390	2.642	0.115	0.003	0.956	51.56	<0.0005	0.180	0.675	0.039	0.846	1.836	0.186	
T-maze working memory	Score (training-1s-15s-30s)	-	2, 3	1, 29	5.318	0.028	0.457	0.504	0.042	0.839	8.336	<0.0005	1.379	0.254	0.070	0.976	1.434	0.238	
	Score (1s-5s-15s-30s)	2F	2, 3	1, 29	6.631	0.015	0.504	0.483	0.681	0.416	6.661	<0.0005	1.442	0.236	0.368	0.776	1.035	0.381	
Reference memory	Score (Blocks 4-14)	2G	4	1, 29	0.995	0.327	2.250	0.144	1.801	0.190	40.05	<0.0005	1.306	0.278	0.336	0.788	0.643	0.581	

Supplementary Table 1c				Repeated-measures ANOVA: Between-subject effects						S. main effect of genotype			
Behavioural pharmacology, within-subject studies				genotype		environment		interaction		RE		HE	
Behaviour: drug	Tested variable	Fig.	df1, df2	F	p	F	p	F	p	Veh	MK	Veh	MK
LMA: MK-801	total distance moved: 0-90 min	1F	1, 29	14.20	0.001	5.334	0.028	4.820	0.036	0.522	0.298	0.054	<0.0005
	total distance moved: 5-30 min	1G	1, 29	17.289	<0.0005	5.613	0.025	1.476	0.234	0.314	0.044	0.052	<0.0005

Supplementary Table 1c, continued				Repeated-measures ANOVA: Within-subject effects							
Behavioural pharmacology, within-subject studies				drug		drug*env		drug*geno		drug*env*geno	
Behaviour-drug	Tested variable	Fig.	df1, df2	F	p	F	p	F	p	F	p
LMA: MK-801	total distance moved: 0-90 min	1F	1, 29	263.0	<0.0005	0.014	0.908	18.418	<0.0005	8.195	<0.0005
	total distance moved: 5-30 min	1G	1, 29	37.204	<0.0005	0.989	0.329	11.714	0.002	1.477	0.234

Analysis of baseline behaviour of *Grin1*$^{\Delta Ppp1r2}$ knockouts compared to controls. Analysis was conducted separately for parameters without *within-subject repetition* using univariate ANOVA (Table 1a), for parameters with *within-subject repetition* using repeated-measures ANOVA (Table 1b), or for the pharmacological test of the effect of MK-801 on locomotor activity using repeated-measures ANOVA (Table 1c). Simple main effects (S. main effect) are used as post-hoc tests in 1a and c, to assess differences of genotype within the two groups with reduced (RE) or high (HE) environmental enrichment (1a), further split up into sub-groups that received vehicle (Veh) or MK-801 (MK) in Table

1c; the stated numbers are *p*-values. The *n*-numbers are not stated as they are identical across tests except for the novel-object recognition test, where 1 RE-control had to be excluded because of lack of interaction with one object in the test phase, the reciprocal social interaction where 2 KO and 2 WT HE-mice were excluded because of repeated aggression, and the PPI-test where 2 controls and 2 knockouts were excluded due to too low startling (< 100); for all other test the numbers are: RE, 7 KO, 6 Ctrl; HE: 9 KO, 11 Ctrl. For reciprocal social interaction, the RE-group is not included in the analysis, because the majority of mice showed repeated aggression. The average age of the cohort at which the experiment was conducted is given in months, and is always identical for the HE and RE groups. For the T-maze analysis, the data from the two test sessions conducted at the stated ages were averaged within each protocol. The MK-801 assay was conducted at an age of 4 months, at the end of the test battery. Where the cell stating df1, df2 is highlighted purple, the degrees of freedom and *p*-value have been adjusted using the Greenhouse-Geisser correction for testing the within-subject effects, because Mauchly's test indicated a violation of sphericity. All other repeated-measures ANOVAs where conducted assuming sphericity, whereby the degrees of freedom are identical for the stated between- and the within-subject analyses.

Abbreviations: env., environment; geno, genotype; Fig., figure panel which displays the analysed data; WSF, within-subject variable in the repeated-measures ANOVA; PPI, pre-pulse inhibition; SNP, spatial novelty preference; LMA, locomotor activity; df, degrees of freedom in ANOVA, F, F-value of the ANOVA, *p*, p-value of the ANOVA.

Supplementary methods

Animals and environmental enrichment

Mice with a BAC-transgenic genomic insertion of *Cre*-recombinase driven by the protein phosphatase 1 regulatory (inhibitor) subunit 2 (or Ppp1r2) promoter (Belforte et al., 2010) (C57BL/6N-Tg(Ppp1r2-cre)4127Nkza/J, Jackson Labs stock number 012686), were crossed to Grin1-2lox mice in which the endogenous NMDAR-subunit *Grin1* is replaced by a homologous gene with loxP-sites flanking exons 11-18 (Niewoehner et al., 2007) (B6.129-Grin1^{tm1Rsp}/Kctt, EMMA stock number EM:09220). Grin1-2lox mice were kindly provided by Peter Seeburg and Rolf Sprengel at the Max-Plack-Institute for Medical Research, Heidelberg, Germany.

Both genetic components are optimized for ablation of NMDA-receptors starting as *early* as possible to capture aspects of NMDAR-hypofunction during development more appropriately: the Ppp1r2-promoter drives expression in PV-interneurons approximately a week earlier (starting around postnatal day (P) 7), than the parvalbumin-promotor itself (which is, however, much more specific to PV-interneurons) (Belforte et al., 2010). The Grin1-2lox line, in turn, features a comparatively short distance between the two loxP-sites of 3.1 kB. Using the same Ppp1r2-*Cre* driver line as used here, it was shown previously, that a shorter distance between loxP-sites leads to stronger schizophrenia-related phenotypes, presumably due to the statistically higher chance of recombination which leads to an earlier NMDAR-ablation on average across the cell population (Belforte et al., 2010). The same publication also described that this mouse line (using a floxed Grin1-line with a distance of 2.1 kb between loxP-sites) shows deficits in pre-pulse inhibition, reciprocal social interaction, nest building, saccharine-preference and spatial short-term memory (spontaneous alternation) and decreased Gad67-expression, although some of these phenotypes were observed after many weeks of social isolation, which started right at the end of weaning (Belforte et al., 2010; Jiang et al., 2013), and therefore might still have interfered with developmental aspects of the disease rather than modelling environment-related stress in the adult brain. Basic physiological measures, including vision, audition, olfaction and motor coordination appeared normal.

For genotyping, PCR-mediated amplification from genomic DNA-samples using primers 5‘ – TGT GTC CCT GTC CAT ACT CAA – 3‘ and 5‘ – AAC ACT GTG GAC CAG GAC TTG – 3‘ resulted in a 325 bp product for the Grin1-wildtype allele and in a 375 bp product for the floxed allele. *Cre*-recombinase was detected with generic primers 5’- CAC CCT GTT ACG TAT AGC CG – 3’ and 5- GAG TCA TCC TTA GCG CCG TA – 3’ resulting in a 330 bp product. Mice with one *Cre* and two floxed-*Grin1* alleles were deployed as $Grin1^{\Delta Ppp1r2}$

knockouts (termed knockouts, KO or *Grin1*$^{\Delta Ppp1r2}$ throughout the manuscript), while *Cre*-negative littermates with usually two and rarely one floxed-*Grin1* allele(s) were used as control mice (termed controls, Ctrl, throughout the manuscript). All mice were genotyped again at the end of the test battery, to confirm the result obtained before testing started.

To study the effect of environmental enrichment/deprivation in adulthood, mice were at first bred and grown in individually-ventilated cages (IVC, Type-II, Blue-Line, Tecniplast, Italy) highly enriched with sizzle nest, a card board house and a card board tube (all from Datesand, UK). At 4-6 weeks of age one sub-group of mice was transferred to IVC-cages enriched with only one pressed cotton pad of ca. 2.5 g (Nestlet^{TM}, Datesand), which represents the standard enrichment in many countries, while another cohort was housed in type-II-long open-top cages enriched in the same way as the breeding cages (sizzle nest, tube, house) to provide rich tactile, auditory and olfactory stimulation (Supplementary Figure 1). The former type of environment is termed *reduced environmental enrichment* (RE), while the latter is termed *high environmental enrichment (*HE*)* throughout the manuscript. All cages contained sawdust. Behavioural testing started 6 weeks after animals had been transferred into those respective environments. That sequence started with assessment of novelty-induced hyperlocomotion at two months of age, continued with tests of anxiety, cognition, social behaviour and anhedonia (in that order, 2-3 months of age) and finished with pre-pulse inhibition and MK801-induced hyperlocomotion before 20 weeks of age.

Using the complete test battery described below, we tested one adult male cohort with 4 subgroups, including 7 knockouts and 6 controls maintained in RE housing condition as well as 9 knockouts and 11 controls maintained in HE housing condition. The average age at testing was between 2 and 4 months for the entire test battery, and it is mentioned for each test in Supplementary Methods below and in Supplementary table 1 above. We tested the mice at this comparatively young age, because it had been reported previously, that after the age of 20 weeks (5 months) the Ppp1r2-*Cre* driver starts expressing in granule cells in the dentate gyrus (Belforte et al., 2010). Mice were group-housed at all times, except when isolation was required during the experiments themselves (i.e. for sucrose preference testing and nest building, which are assessed over multiple consecutive days). Behavioural testing was conducted during the light phase, except for assessments of sucrose preference and nest building. Experiments that involved manual scoring or intense handling (Y-maze, T-maze, Plus-maze, novel object recognition, social tests) were conducted blind to genotype. All experiments conformed to the Animal (Scientific Procedures) Act 1986, UK, and the Local Ethical Review Committee at the University of Oxford.

Novelty-induced locomotor activity

To assess novelty-induced hyperactivity mice were placed into novel clear plastic cages (l 43 cm, w 22 cm, h 20 cm) containing clean sawdust, and locomotor activity was measured by infrared beam breaks (San Diego Instruments, San Diego, CA, US) for 2 hours in 5-minute time bins. The test was conducted twice, at the age of 2 and 4 months, respectively.

Spatial novelty-preference (Y-maze, spatial short-term memory)

Spatial novelty preference was assessed at the age of 2 months using a clear Perspex Y-maze (each arm: w 8 cm, l 30 cm, h 20 cm) as previously described (Sanderson et al., 2007). To promote exploratory behaviour the maze was scattered with a mixture of clean and dirty sawdust (3:1) from the cages of unfamiliar mice of the same sex. In the sample trial mice were placed in the start arm and allowed to explore the maze for 5 minutes with either the left or right goal arm blocked off by a non-transparent black door (counterbalanced within genotype). Mice were then removed from the maze for an intra-trial interval (ITI) of 1 minute during which the sawdust was mixed and re-distributed around the maze and the divider removed. In the choice phase mice were given 2 minutes to explore the entire Y maze. The location of the mouse was tracked with ANY-maze (San Diego Instruments). The preference for the novel goal arm was calculated as the ratio of the time spent in the novel arm divided by the time spent in both choice arms combined.

Novel object recognition (object short-term memory)

This task was done as described previously (Bygrave et al., 2016; Sanderson et al., 2011). Mice (3 months of age) were habituated to a dark grey open field (40 x 40 cm, 25 cm wall height) 3 times for 5 min per day for 2 days prior to testing. On the test day, mice were exposed to two identical copies of object X for 10 minutes. Mice were then removed from the open field for 2 min, during which the initial objects were replaced by an identical copy of object X and a novel object Y. Mice were then reinserted into the open field and left to explore for 5 min. Direct exploration of the objects was manually scored by the experimenter blinded for genotype, and videos were recorded using ANY-maze (San Diego Instruments). Objects and the arena were cleaned with 70 % ethanol and water between animals. The identity of the first object (X or Y) and its position in the arena were counterbalanced within each genotype. The preference for the novel object was calculated as the ratio of the time spent with the novel object divided by the time spent with both objects combined. The same ratio, but using the number of contacts, was used as a secondary measure. Beverage containers (cans, plastic and glass bottles) were used as objects and chosen to ensure a differentiation between shape, colour, surface pattern/texture and material.

Rewarded alternation on the T-maze

Rewarded alternation was tested in a wooden elevated T-maze painted grey (start arm: w 10 cm, l 47 cm; goal arms: w 10cm, l 35cm; wall-height 10 cm; elevated 100 cm above ground) with metal food wells fixed to the end of each goal arm. Prior to testing animals were put on a food-restricted diet to limit them to 85-90 % of their free-feeding weight. Mice were then habituated to the maze and the condensed milk reward (mixed 50:50 with drinking water), initially in groups and then individually. During training and testing, each trial consisted of a sample and a choice run. In the sample run mice were placed in the start arm facing the experimenter and forced into one of the goal arms to gain a reward. The forced direction was randomly assigned, with no more than three consecutive trials in the same direction, and overall equal numbers of left and right allocations. Mice were then removed from the maze for a defined intra-trial interval (1 s, 5 s or 15 s, as indicated), during which the door from the second goal arm was removed. For the choice run, mice were returned to the maze start arm facing the investigator and allowed to make a free choice between both goal arms, of which the previously blocked (unvisited) one was rewarded. A decision was counted when the mouse had crossed the T-junction and all four paws were within a goal arm. The selection of protocols was determined according to a scheme, which had revealed a subtle deficit at the short (1s) delay protocol in two studies with a selective NMDA-receptor knockout in PV-interneurons previously (Bygrave et al., 2016; Carlen et al., 2012): at first, mice received 3 days of training with an inter-trial interval (ITI) of 4-6 min (round-robin or “blocked” regime) and a delay of 5 s. Subsequently, a massed design was applied with a minimal (1s) delay and an ITI of 45 s for 1 d. On the two final days, a blocked design was used again with a delay of15 s (day 5) and then 30 s (day 6) to challenge the maintenance of the memory. In order to ensure the robustness of the findings the whole sequence was repeated one month later, and the results from both sessions were averaged within each protocol for the final analysis. Thereby, testing was conducted at the ages of 2 and 3 months, for the first and the second session, respectively.

Spatial reference learning

Food deprivation, maze adaptation and reward were as described for the rewarded alternation task above. An elevated grey wooden plus maze with 10 cm wall height and 10 cm inner arm width was used; arm-length was 35 and 40 cm for goal and start arms, respectively. For each mouse, counterbalanced for genotype, one goal arm location (as defined by allocentric extra-maze cues in the room) was assigned as rewarded. The maze was rotated by 180° once per day to prevent association of subtle inner-maze cues with the reward. On every trial the start arm was pseudo-randomly chosen from the two start arms of

the maze in order to prevent the subjects from using an egocentric strategy to solve the task. Mice were trained to a criterion of correct choices in 17/20 (85 %) consecutive trials. Subsequently mice were subjected to a block of 10 trials, during which reward was delivered after the choice was made, to ensure they were not solving the task simply by smelling the reward. Testing was started in the 17th week of age on average, i.e. as the mice turned 4 months old.

Sucrose preference

Mice were individually housed for this test, and the test was conducted during the dark phase. Cages contained two water bottles, placed on the same side of the cage. The test was conducted over three consecutive nights. During the first night, both bottles contained normal drinking water. The contents of one bottle were replaced with 10 % sucrose (w/w; Sigma-Aldrich, Germany) in drinking water for the subsequent nights. During day time, the sucrose-containing bottle was removed and only the water bottle was left in the cage. The location of the sucrose-containing bottle was counterbalanced within each genotype, but not altered from one testing day to the next. Consumption between ca. 7 pm and 8 am was measured for each bottle on each day and the preference for sucrose calculated as the ratio of consumption of sucrose-solution to total liquid consumption. Testing was conducted at an age of 4 months.

Nest building

Assessment of nest building was performed overnight as previously described (Deacon, 2006), immediately after the last night of sucrose-preference assessment. Mice were single housed in a cage containing sawdust and 3 g Nestlet^{TM} material (Datesand). All enrichment items were removed beforehand and food pellets were provided in the saw dust instead of the food hopper. Unused Nestlet^{TM} material was weighed the next morning and the quality of the nest was rated on a 5-point scale by two observers blind to genotype according to established scoring guidelines (Deacon, 2006). Intermediate scores were assigned in cases of discrepancy between the weight of unused Nestlet^{TM} material and overall appearance of the nest. Testing was conducted at an age of 4 months.

Non-reciprocal social interaction (3-chamber test)

Testing was conducted in a 3-chamber apparatus as described previously (Moy et al., 2004), at low light levels (ca. 1 lux). CD1-mice of the same sex and weight (± 10%) were used as stimulus mice and were habituated to the apparatus and the small enclosures over 3 days

before testing commenced. The test consisted of four phases, between which animals were enclosed in the central chamber but not removed from the apparatus: (i) habituation to the central chamber of the apparatus (5 min), (ii) habituation to all three empty chambers (10 min), (iii) social exposure (10 min) and (iv) social memory (10 min). During the social exposure phase a test mouse was presented with two metal cage enclosures in the far corners of the peripheral chambers, one of which contained a CD1 stimulus mouse. For the social memory phase, the empty enclosure was replaced by a third enclosure containing a CD1 stimulus mouse from a different home cage. Within each genotype and enrichment group, the locations of the stimulus mice were counterbalanced with respect to their location in phase (iii) and phase (iv). In phase (iv), counterbalancing included a transfer of the enclosure with the familiar mouse to the other chamber (which did not contain a mouse in phase (iii)), while its original place was taken by the enclosure with the new mouse for 50 % of the mice in each sub-group. Interactions were scored as entries into a 2 cm circumference of the metal cages by automated tracking in ANY-Maze (San Diego Instruments). Entries into this interaction zone were registered if 30 % of the animal's body is present within the zone, and exits were scored if less than 20 % was present in the zone. Testing was conducted at an age of 3 months.

Reciprocal social interaction (repeated direct interaction between stimulus and test mouse in an open field)

Testing was conducted at low light levels (ca. 10 lux) in the same Type III Perspex cages as were used for the locomotor activity assessment (see above), using a fresh cage with clean saw dust for every mouse. The protocol was identical to the one applied in this mouse strain before to assess social interaction and memory (Belforte et al., 2010). Juvenile, same-sex, unfamiliar CD1-mice (3-4 weeks of age) were used as stimulus mice. The test mice – but not the stimulus mice - were habituated to the test cages for 1 h. Immediately afterwards, a CD1-stimulus mouse was added to the cage for 1 min, then removed again for 9 min, before a second 1 min-exposure. This cycle continued for a total of 5 exposures and subsequent 9 min intervals, where the test mouse was alone in the cage. Immediately afterwards, a yet unfamiliar CD1-stimulus mouse was added to the test cage for 1 min, after which the testing was concluded. If test-mice conducted direct attacks in more than one exposure period, they were scored as aggressive. The testing was then terminated prematurely to protect the stimulus mouse as this usually indicated, that mice would not interact in a non-aggressive manner anymore in subsequent exposures. Social interactions were recorded with CCTV cameras and the number and total duration of direct social interactions was scored blind to genotype. Such interactions included sniffing, direct tactile or whisker-mediated contact but not aggression or sexual behaviour. Testing was conducted at the age of 4 months.

Pre-pulse inhibition (PPI)

Mice were inserted into transparent plastic tubes inside startle-response test cabinets (San Diego Instruments). After an adaptation period of 5 min at a background white noise of 65 dB, mice were subjected to a sequence of white-noise tones which started and ended by a series of five 120 dB startle-pulses, and contained 10 presentations each of startle-pulses (120 dB), pre-pulses at four levels (69 dB, 73 dB, 77 dB, 81 dB), and combinations of each pre-pulse with the startle-pulse (PPI-trials) in between. Presentations were randomized for type and occurred at random inter-trial-intervals of 10-20 s (average: 15 s). Pre-pulses lasted 20 ms, startle-pulses 40 ms, and they were separated by 100 ms intervals in PPI-trials. The pre-pulse inhibition (PPI) was calculated for each PPI-trial individually using the average startle-response from those ten 120 dB presentations interspersed with other trials (startle-response) as the baseline and the response from the respective PPI-trial (PPI-response) according to:

PPI = 100 – (100 * PPI-response/startle-response)

PPI-values were then averaged for each pre-pulse level.

The habituation to the startle pulse was calculated from the averages of the first five (startle(start)) and the last five (startle(end)) startle-responses evoked by the respective 120 dB presentations flanking the remaining sequence. Mice that had a startle-response of less than 100 were excluded from the dataset, as such low levels indicate hearing problems; this affected 2 knockouts and 2 controls (whereby one control mouse was from the standard housing condition, the other three from the enriched group); the average startle response (± S.D.) in the complete cohort, incl. excluded mice, was 251 ± 167. Testing was conducted at an age of 4 months.

MK-801-induced locomotor activity

MK-801-induced locomotor activity was assessed at the end of the test battery (4 months of age) in the same Type III cages as used for novelty-induced locomotion (see above) and were cleaned and filled with fresh sawdust before each run. Each animal received two runs, of which one included a vehicle-injection and the other an injection of MK-801 (within-subject design), on different days. As done previously (Belforte et al., 2010; Bygrave et al., 2016), each run started with a 30 min habituation period, in which mice explored the cage for the first time and displayed novelty-induced hyperlocomotion; immediately afterwards either vehicle or a dose of 0.2 mg/kg MK-801 ((+)-dizocilpine maleate, Tocris, UK) as a solution of 0.05 mg/ml in sterile saline vehicle was injected i.p. using an injection volume of 4 μl/g and

locomotor activity was monitored for another 90 min. Locomotor-activity was measured from beam-breaks in the photo-cell cages, and animals were video-recorded during testing after drug injection.

Analysis

Time-series and within-subject data were analysed using repeated measures ANOVA. Other data with multiple independent variables were assessed with univariate ANOVA followed by simple main effects post-hoc tests for effects of genotype within sub-groups. Data-sets with a single independent variable were analysed by *t*-tests or Mann-Witney-U-Test (abbr. M.W.U-Test) as appropriate. Two-sided tests were used throughout. Significance level was $P < 0.05$ throughout. Except for locomotor interval graphs, data are plotted with error bars as 95 % confidence intervals placed symmetrically around the mean for ease of pairwise and 1-sample comparisons. Error-bars in locomotor interval plots represent S.E.M. for clarity of presentation.

Supplementary References

Belforte, J. E., Zsiros, V., Sklar, E. R., Jiang, Z., Yu, G., Li, Y., et al. (2010). Postnatal NMDA receptor ablation in corticolimbic interneurons confers schizophrenia-like phenotypes. *Nat Neurosci* 13, 76–83.

Bygrave, A. M., Masiulis, S., Nicholson, E., Berkemann, M., Sprengel, R., Harrison, P., et al. (2016). Knockout of NMDA-receptors from parvalbumin interneurons sensitizes to schizophrenia-related deficits induced by MK-801. *Transl. Psychiatry*.

Carlen, M., Meletis, K., Siegle, J. H., Cardin, J. A., Futai, K., Vierling-Claassen, D., et al. (2012). A critical role for NMDA receptors in parvalbumin interneurons for gamma rhythm induction and behavior. *Mol Psychiatry* 17, 537–548.

Deacon, R. M. (2006). Assessing nest building in mice. *Nat. Protoc.* 1, 1117–1119. doi:10.1038/nprot.2006.170.

Jiang, Z., Rompala, G. R., Zhang, S., Cowell, R. M., and Nakazawa, K. (2013). Social Isolation Exacerbates Schizophrenia-Like Phenotypes via Oxidative Stress in Cortical Interneurons. *Biol. Psychiatry* 73, 1024–1034. doi:10.1016/j.biopsych.2012.12.004.

Moy, S. S., Nadler, J. J., Perez, A., Barbaro, R. P., Johns, J. M., Magnuson, T. R., et al. (2004). Sociability and preference for social novelty in five inbred strains: an approach to assess autistic-like behavior in mice. *Genes Brain Behav.* 3, 287–302. doi:10.1111/j.1601-1848.2004.00076.x.

Niewoehner, B., Single, F. N., Hvalby, ?, Jensen, V., Meyer zum Alten Borgloh, S., Seeburg, P. H., et al. (2007). Impaired spatial working memory but spared spatial reference memory following functional loss of NMDA receptors in the dentate gyrus. *Eur. J. Neurosci.* 25, 837–846. doi:10.1111/j.1460-9568.2007.05312.x.

Sanderson, D. J., Gray, A., Simon, A., Taylor, A. M., Deacon, R. M. J., Seeburg, P. H., et al. (2007). Deletion of glutamate receptor-A (GluR-A) AMPA receptor subunits impairs one-trial spatial memory. *Behav. Neurosci.* 121, 559–569. doi:10.1037/0735-7044.121.3.559.

Sanderson, D. J., Hindley, E., Smeaton, E., Denny, N., Taylor, A., Barkus, C., et al. (2011). Deletion of the GluA1 AMPA receptor subunit impairs recency-dependent object recognition memory. *Learn. Mem.* 18, 181–190. doi:10.1101/lm.2083411.
